# Stigmatization of people with mental illness – a matter of milieu-specific worldviews? Results from a population-based survey in Germany

**DOI:** 10.3389/fpsyt.2024.1501194

**Published:** 2025-01-28

**Authors:** Jenny Spahlholz, Eva Baumann, Sven Speerforck, Christian Sander, Matthias C. Angermeyer, Georg Schomerus

**Affiliations:** ^1^ Department of Psychiatry and Psychotherapy, University of Leipzig Medical Faculty, Leipzig, Germany; ^2^ Department of Journalism and Communication Research, Hanover University of Music, Drama, and Media, Hanover, Germany; ^3^ Department of Psychiatry and Psychotherapy, University of Leipzig Medical Center, Leipzig, Germany; ^4^ Center for Public Mental Health, Gösing am Wagram, Austria

**Keywords:** stigma, depression, schizophrenia, mental illness, milieu

## Abstract

**Background:**

Despite numerous awareness campaigns and anti-stigma programs, people with mental illness, particularly schizophrenia, are still stigmatized. Although the society is both cause and solution, societal-level conditions, such as society’s customs and policies that legitimize and perpetuate stigmatization is often neglected. We used a milieu approach to investigate how shared social, cultural and political orientations and expectations are associated with manifestations of the mental-illness related stigma.

**Methods:**

We analyzed cross-sectional data from 3,042 adults aged ≥18 years from a national vignette-based representative survey on the stigma of mental illness in Germany from 2020. For milieu classification, we used an established population segmentation tool based on values and political preferences. Two stigma measures associated with the stereotype and status loss/discrimination components were assessed (i.e., the Social Distance Scale and a list of well-known stereotypes associated with depression or schizophrenia). Descriptive analyses and one-way ANOVAs with *post-hoc* pairwise contrasts between milieu groups were used to evaluate agreement on stereotypes and the desire for social distance towards people with depression or schizophrenia.

**Results:**

Negative stereotypes about people with a depression (i.e., beliefs about being weak-willed) and schizophrenia (i.e., beliefs about dangerousness) tended to be more common in milieu groups leaning more toward the authoritarian pole. Milieu groups with a more liberal attitude on the socio-cultural dimension further expressed a lower desire for social distance towards people with depression (p<0.001). However, the extent of differentiation between the milieu groups was less pronounced regarding the desire for social distance towards people with schizophrenia than towards people with depression.

**Conclusion:**

Our findings suggest that socio-cultural and socioeconomic dimensions of the society can be used for both describing heterogeneous societies and illuminating the underlying social structure of stigma. In addition to making blind spots more visible (i.e., schizophrenia), milieu-specific knowledge could be useful in deciding which intervention components are most appropriate for which milieu groups and how to apply them successfully.

## 1 Introduction

Since the late 1990s, the dual burden of mental illness and mental illness-related stigma has become globally more aware. This may largely be contributed to the efforts of international organizations such as the World Health Organization (WHO), the World Psychiatric Association (WPA), the World Association for Social Psychiatry and several nongovernmental organizations (1). Educational programs on schizophrenia (i.e., the Open-the-Doors program) were WPAs first step in what would become a major international effort to combat stigmatization of people with schizophrenia (2, 3). A short time later, the WHO systematically addressed the stigma related to mental illness and its consequences through awareness raising and advocacy efforts. The recognition of stigma as an important barrier to appropriate treatment (i.e., recognized as such for the first time in the World Health Report, 2001) and the implementation of global anti-stigma awareness campaigns (i.e., implemented as such for the first time by the World Health Day, 2001) were important milestones on this path (4–6). Stimulated by these developments, numerous programs and campaigns aimed at reducing stigmatization have been undertaken across the world (7–9). But fighting stigma has turned out as a multifaceted, difficult, and yet unresolved task on different societal levels.

Large-scale anti-stigma programs (i.e. England’s Time to Change programme) are found to have small to moderate long-term effects (10). Furthermore, time-trend studies on changes of stigma in the U.S. and Germany (8, 11) equally found that stigmatizing attitudes are specific to different mental health conditions by providing evidence of significant decreases in public stigma toward depression, but not for schizophrenia or alcohol dependence.

These findings have provoked ideas on rethinking the stigma concept and on retooling reduction strategies. There are calls for addressing more intensively structural aspects of the mental illness stigma (11) by recognizing societal-level conditions that legitimize and perpetuate stigmatization (12). Stigma is a social phenomenon rooted in social structures (13). The decision to stigmatize an individual is not an individual decision; rather, it is embedded in social structures in which people live.

Consequently, there is a need to focus on both discriminatory social structures (e.g., laws and policies) and on existing cultural norms that make it justifiable to devalue certain identities/statuses (12, 14). Aside from cultural variations in the mental illness stigma (15), we argue that there is a need to analyze differences within societies more intensively. Due to growing migration worldwide, western societies have become more ethnically and culturally diverse (16). And in the light of increasing and polarizing social conflicts, tendencies of social division (i.e., the experience of living in different worlds or bubbles) are often discussed. German society is far away from being divided into ‘separate bubbles’ (17). However, societies are made up of different social groups that may, of course, represent different social, cultural, and political values.

Values are intertwined with attitudes and behavior and might therefore be reflected in both attitudes and behavior of people. (18). A closer look at certain social groups might show differences between these groups in the way they think and behave towards mental illnesses. Traditional analyses are largely limited to the question of how certain attitudes or discriminatory behavior are distributed in the population as a whole, but not across individual social groups.

And, beyond that, existing subgroup analyses on sociodemographic characteristics provide limited insight into stigma in general, as well as the differences in stigma between depression and schizophrenia (11). In the present study, we therefore use a milieu approach to shed light on broader social strata. In addition to the objective social situation, measured by income or education, attitudes and basic orientations, the mentalities of people are considered (19). Looking at stigmatization through a ‘milieu lens’ offers an opportunity for studying how social, cultural, and political realities are related to mental-illness stigma. It recognizes value orientations and expectations about society as lenses on what is important for people (e.g., liberalism, traditionalism, achievement-orientation). In this study, we apply this framework to the sociological understanding of the stigma associated with mental illness, aiming at providing a better understanding of societal-level conditions beyond stigmatization.

## 2 Materials and methods

### 2.1 Study design and sample

We utilized data collected from the fourth wave of a national trend survey (Evolutions2020) with a strict focus on changes in public attitudes towards people with mental illness in Germany (8). The long-term perspective is not considered in the present analysis, as in this wave of data collection the milieus have been surveyed for the first time. Overall, 3,042 people from a probability-based sample of ≥18 years old German provided answers in a face-to-face interview (84,7%) or a self-administered questionnaire (15.3%) which was implemented as an online survey due to COVID-19 restrictions, resulting in a response rate of 57.1%.

Regarding sociodemographic data, our sample was highly comparable to the German population in terms of age and gender, while respondents with a higher educational level (more than 10 years of schooling) were slightly underrepresented (see **Supplementary Table 1**). An external market and social research institute (USUMA, Berlin, Germany) was commissioned with carrying out the data collection for the study; all respondents provided verbal or written consent to participate and received written information about handling of their data and their right to withdraw from the study at any time. The study was approved by the review board of Greifswald University Medical Center (BB 195/18).

### 2.2 Vignettes

Each interview began with the presentation of an unlabeled vignette (German language), describing the symptoms of an individual meeting the DSM-II-R criteria for depression or schizophrenia without mentioning the diagnosis. Respondents were assigned at random to one of four vignettes, varying in symptoms (schizophrenia or major depressive disorder) and gender (male or female). Of the total number of respondents, 1,530 received the depression vignettes, and 1,512 received the schizophrenia vignettes. Respondents were then asked to respond to the vignette on a number of rating scales in German language (also with the following two scales).

### 2.3 Stereotypes

We presented a list of eleven adjectives and asked respondents to rate their level of agreement with the items on a five-point Likert scale from ‘strongly agree’ (1) to ‘strongly disagree’ (5) (20). According to research on stereotypes about people with mental illnesses (21, 22), analyses presented here address only those adjectives that have revealed as most relevant for people with a depression (i.e., beliefs about being weak-willed) or people with a schizophrenia (i.e., beliefs about dangerousness).

### 2.4 Desire for social distance

We used the Social Distance Scale (SDS) developed by Link et al. (23) to assess respondents’ willingness to accept the person described in the vignette in seven hypothetical situations like working together or having as a neighbor (i.e., as an measure of discrimination). Responses were given on a five-point-Likert scale from ‘very likely’ (1) to ‘very unlikely’ (5) (higher scores indicate stronger desire for social distance). Cronbach’s Alpha was .92.

### 2.5 Milieu groups

The milieu group classification applied in the present study represent a two-dimensional model that maps individual basic values and political attitudes along a socio-cultural and a socioeconomic axis. Originally developed in a study by the Friedrich Ebert Foundation (24), the milieu typology was validated and adapted to changing social realities by Müller-Hilmer and Gagné (25). The present study is based on this adapted typology of nine milieus, which was already applied by the authors of the present study to evaluate milieu-specific help-seeking behavior due to emotional problems. Full details of the methodology have been reported elsewhere (26), details of the sociodemographic characteristics of the milieu groups are included as a supplement in Spahlholz et al. (26).

On a two-axis chart with a vertical socio-cultural dimension (libertarian versus authoritarian values) and a horizontal socioeconomic dimension (market liberalization versus a more regulated economy), four out of nine milieu groups can be positioned in the left quadrants. These milieu groups endorse a more regulated economy and mainly the younger and high educated *cosmopolitan intellectuals* and the *committed citizenship* strongly adopt liberal values. More authoritarian along the socio-cultural axis (but also with pro-government regulation preferences), *the market sceptics*, consisting of a large proportion of elderly people (above 60 years) exhibit high levels of distributional concerns and market skepticism. While the much more authoritarian and younger *participation-oriented* express strong demands in general welfare state principles toward their own nation whilst refusing policies related to European solidarity.

Four further milieu groups can be positioned on the right of the socio-economic axis. Respondents of these milieu groups support a free-market economy, which they consider beneficial to all people (i.e., *the social market optimists*). Also, in favor of market liberalization, but closer to the authoritarian pole, *the conservatives, the performance-oriented*, and *the individualists* can be positioned. They value performance-related compensation and gratification (*the conservatives*), have strong preferences for promotion of elites (*the performance-oriented*) or adopt more clearly anti-migration, anti-EU, and anti-globalization positions (*the individualists*). In the middle of socioeconomic axis but also close to the authoritarian pole, one milieu group can be positioned (*the disappointed*).

### 2.6 Analyses

For the descriptive analyses, means, standard deviations, and percentages were used. Regarding the *stereotypes*, we collapsed the responses of ‘strongly agree’ and ‘agree’ into one category. Overall percentage of agreement was calculated as the number of observed agreements divided by the number of possible agreements (separated by milieu groups). Regarding the *SDS*, mean scores and standard deviations were calculated for all milieu groups, higher scores indicate stronger desire for social distance. Cronbachs’s alpha for the SDS was used to evaluate the reliability of internal consistence. We used one-way analyses of variance (ANOVAs) with *post hoc* pairwise contrasts to test differences in the desire of social distance towards people with a depression or a schizophrenia between the nine milieu groups. Variables were tested for normal distribution and homogeneity of variance. Although, the normality assumption was not fulfilled, a parametric method was used in the analyses. We therefore refer to the large portion of the literature on robustness of F-test for ANOVA to non-normality (27–29) and utilized a bootstrapping with 1,000 resamples. The significance level was set as p < 0.05, and all data analyses were conducted using Stata (16.1, StataCorp LLC, College Station, TX).

## 3 Results

### 3.1 Descriptive results from the assessment of stereotypes

For the depression vignette, 41.7% of the respondents of *the participation-oriented* agreed with the statement that the individual described in the vignette is weak-willed, followed by 37.8% of *the disappointed*, and 31.6% of *the performance-oriented milieu group.* The statement was further agreed by one quarter of the respondents of *the conservatives (*26.8%), *the market sceptics* (25.6%), and *the individualists* (21.3%). Within the more liberal milieu groups, agreement rates for the ‘weak-willed’-statement ranged between 17.2% (*the cosmopolitan intellectuals*) and 21.5% (*the committed citizenship*).

Regarding the schizophrenia vignette, every second respondent of *the participation-oriented milieu group* (50.9%) agreed with the dangerousness-statement. The overall levels of concerns on this issue were also quite high within the remaining authoritarian milieu groups, with about one third of the respondents of *the disappointed* (34.6%), *the conservatives* (33.5%), *the performance-oriented* (31.1%), and *the individualists* (29.6%) perceiving people with a schizophrenia as dangerous. Within the more liberal milieu groups, 19.0% of *the social market optimists* viewed the individual in the vignette as dangerous, followed by 21.2% of *the cosmopolitan intellectuals*, 22.5% of *the committed citizenship, and* 24.1% of *the market sceptics*. Agreement with the statements are listed in **Table 1**.

**Table 1:** Agreement with the statements (in %).

	Agreement with the weak-willed statement		Agreement with the dangerousness statement	
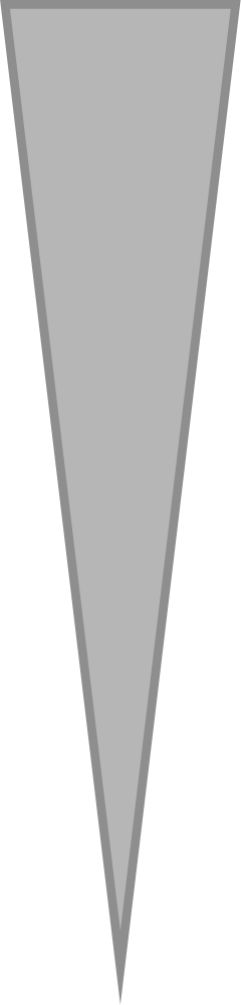	participation-oriented	41.7%	participation-oriented	50.9%
	disappointed	37.8%	disappointed	34.6%
	performance oriented	31.6%	conservatives	33.5%
	conservatives	26.8%	performance-oriented	31.1%
	market sceptics	25.6%	individualists	29.6%
	committed citizenship	21.5%	market sceptics	24.1%
	individualists	21.3%	committed citizenship	22.5%
	social market optimists	19.8%	cosmopolitan intellectuals	21.2%
	cosmopolitan intellectuals	17.2%	social market optimists	19.0%

### 3.2 One-way ANOVA test results for the SDS scale

**Figure 1:**
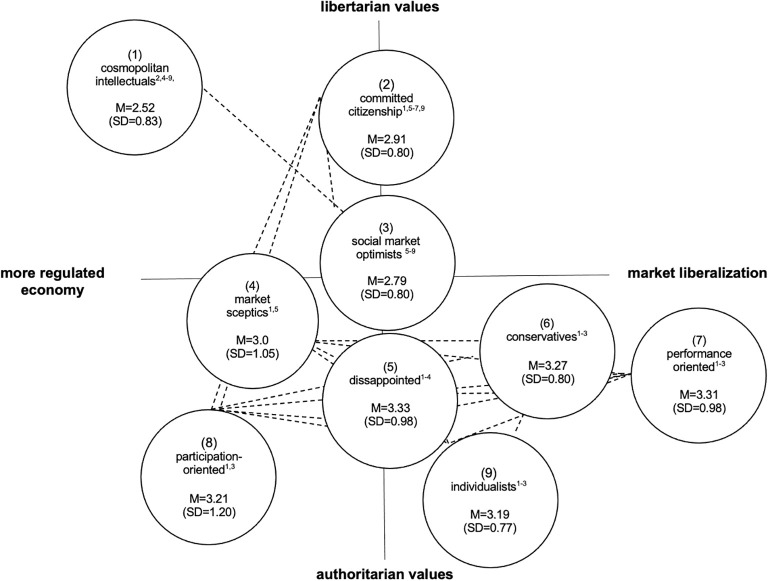
Mean ratings (M) of public's desire for social distance from people with a depression, separated by milieu groups (Adapted from 25). Dotted lines represent groups with similar levels (i.e. differences below significance) of social distance; numbers in parentheses () represent milieu groups, superscript-numbers in circles represent significant mean differences.

The desire for social distance from people with depression differed statistically significant for the different milieu groups with *F*(8, 1517) = 17.79, *p* < 0.001. As illustrated in **Figure 1**, mean scores on the SDS ranged from 2.52 (SD=0.83; *the cosmopolitan intellectuals)* to 3.33 (SD=0.98; *the disappointed*). The mean score of *the cosmopolitan intellectuals* differed significantly from the mean scores of all other milieu groups, except for *the social market optimists* (M=2.79, SD=0.80), constituting the second lowest overall mean score on the SDS. The mean score of *the social market optimists* was also significantly lower than the mean scores of milieu groups that are leaning more towards the authoritarian pole, with exception of the mean score of *the market sceptics* (M=3.0, SD=1.05). Within the more liberal milieu group, the mean score of *the committed citizenship* differed significantly from that recorded for *the cosmopolitan intellectuals* (2.91 vs. 2.52).

Within the more authoritarian milieu groups, we found statistically significant differences in the SDS mean scores between *the market sceptics* and *the disappointed* (3.0 vs. 3.33). **Table 2** summarizes the results of the *post hoc* pairwise contrasts.

**Table 2:** Statistically significant mean differences across Milieu groups for the social distance depression scores (results of pairwise contrasts from one-way ANOVA after bonferroni adjustment) with 95% confidence intervals (CI).

		Contrast (CI)	*p*
cosmopolitan intellectuals	committed citizenship	0.39 (0.10; 0.67)	<0.001
	market sceptics	0.47 (0.17; 0.77)	<0.001
	participation-oriented	0.80 (0.38; 1.21)	<0.001
	disappointed	0.81 (0.53; 1.09)	<0.001
	conservatives	0.75 (0.45; 1.05)	<0.001
	individualists	0.66 (0.40; 0.93)	<0.001
	performance oriented	0.78 (0.44; 1.13)	<0.001
		
committed citizenship	disappointed	0.42 (0.14; 0.70)	<0.001
	conservatives	0.36 (0.07; 0.66)	<0.01
	individualists	0.28 (0.01; 0.54)	<0.001
	performance oriented	-0.40 (-0.75; -0.05)	<0.01
		
social market optimists	disappointed	0.55 (0.25; 0.84)	<0.001
	conservatives	0.49 (0.17; 0.80)	<0.001
	performance oriented	-0.52 (-0.88; -0.16)	<0.001
	participation-oriented	0.54 (0.11; 0.96)	<0.01
	individualists	-0.42 (-0.68; -0.12)	<0.001
		
disappointed	market sceptics	0.34 (0.04; 0.63)	<0.01

**Figure 2:**
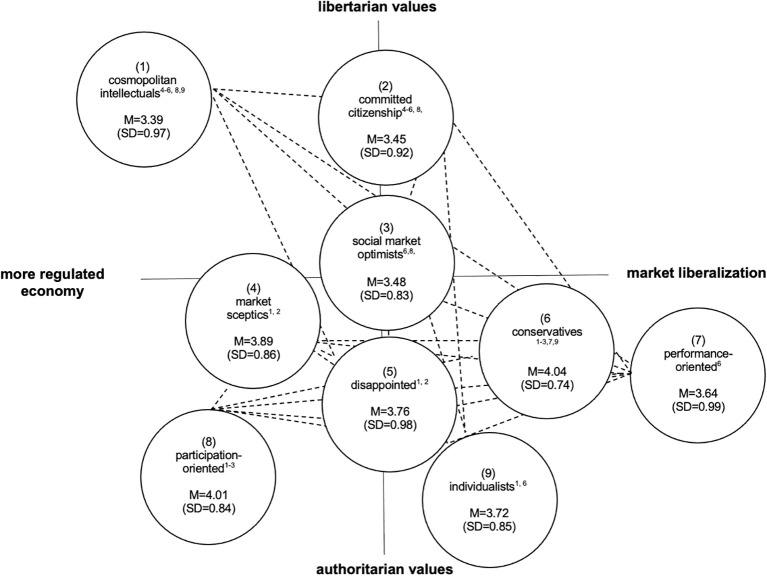
Mean ratings of public's desire for social distance from people with a schizophrenia, separated by milieu groups (Adapted from 25). Dotted lines represent groups with similar levels (i.e. differences below significance) of social distance; numbers in parentheses () represent milieu groups, superscript-numbers in circles represent significant mean differences.

For the schizophrenia vignette, we found significant mean differences in total scores of the SDS across the nine milieu groups with *F*(8, 1492) = 11.23, *p* < 0.001. As illustrated in **Figure 2**, mean scores on the SDS ranged from 3.39 (SD=0.97; *the cosmopolitan intellectuals)* to 4.04 (SD=0.74; *the conservatives*). We found no differences in the SDS mean scores within more liberal milieu groups (i.e., *the cosmopolitan intellectuals, the committed citizenship*, and *the social market optimists)*, but the mean scores differed significantly between the more liberal milieu groups and most milieu groups that are leaning more towards the authoritarian pole. *The cosmopolitan intellectuals* and *the committed citizenship* milieu groups had significantly lower mean scores than *the conservatives* (M=4.04, SD=0.74)*, the participation-oriented* (M=4.01, SD=0.84)*, the market sceptics* (M=3.89, SD=0.86) and *the disappointed* (M=3.76, SD=0.98). The mean score of *the cosmopolitan intellectuals* further differed significantly from the mean score of *the individualists* (M=3.72, SD=0.85). No differences in the mean scores were found between *the cosmopolitan intellectuals* and *the performance-oriented* as well as between *the committed citizenship* and *the performance-oriented* and between *the committed citizenship* and *the individualists.*

The desire for social distance from people with schizophrenia was also lower in *the social market optimists* (M=3.48, SD=0.83) compared to *the conservatives* (M=4.04, SD=0.74) and *the participation-oriented* (M=4.01, SD=0.84). No differences in the mean scores were found between *the social market optimists* and *the market sceptics*, *the social market optimists* and *the disappointed*, *the social market optimists* and *the performance-oriented* as well as between *the social market optimists* and *the individualists*.

Within the more authoritarian milieu groups, we found statistically significant pairwise comparison between *the conservatives* and *the performance oriented* (4.04 vs. 3.64) and between *the conservatives* and *the individualists* (4.04 vs. 3.72). **Table 3** summarizes the results of the *post hoc* pairwise contrasts.

**Table 3:** Statistically significant mean differences across Milieu Groups for the social distance schizophrenia scores (results of pairwise contrasts from one-way ANOVA after bonferroni adjustment) with 95% confidence intervals (CI).

		Contrast (CI)	*p*
cosmopolitan intellectuals	market sceptics	0.50 (0.18; 0.82)	<0.001
	participation-oriented	0.62 (0.18; 1.06)	<0.001
	disappointed	0.36 (0.05; 0.67)	<0.05
	conservatives	0.65 (0.34; 0.96)	<0.001
	individualists	0.33 (0.04; 0.65)	<0.05
committed citizenship	disappointed	0.30 (0.01; 0.58)	<0.05
	conservatives	0.59 (0.31; 0.87)	<0.001
	market sceptics	0.44 (0.14; 0.73)	<0.001
	participation-oriented	0.56 (0.14; 0.98)	<0.01
social market optimists	conservatives	0.56 (0.26; 0.87)	<0.001
	participation-oriented	0.53 (0.10; 0.97)	<0.01
conservatives	performance oriented	0.41 (0.07; 0.75)	<0.01
	individualists	0.33 (0.06; 0.60)	<0.01

## 4 Discussion

We analyzed the interrelation of stigmatization of people with mental illness and the value-world in which people live, think, and act. At this broader, milieu level, our results indicate differences between perceptions and attitudes towards people with depression and schizophrenia among nine milieu groups. Attributions of ‘weakness of will’ and ‘dangerousness’ were common, especially within milieu groups that are leaning more towards the authoritarian pole. We identified at least three out of nine milieu groups with nearly or more than one third of the respondents’ perceiving people with a depression as weak-willed, respectively people with a schizophrenia as dangerous.

This is in accordance with previous studies showing a high agreement with these statements. A review article conducted by Jorm et al. (21), for example, reported prevalence rates of the belief in dangerousness for early and chronic schizophrenia from 20.4% to 60%. While attitudes about depression have improved in the past decades (8, 11), research shows that attributions of personal weakness are still present in the public mind with approval rates of approximately 43% (22, 30)]. Moreover, we identified one milieu group that stands out particularly negatively (i.e., *the participation-oriented milieu group*) with high levels of agreement for both statements and potential preferences in creating or encouraging ‘us-versus-them’ relations. Regarding political and social attitudes, respondents of the *participation-oriented milieu group* expressed strong demands in general welfare principles towards their own nation whilst refusing policies related to European solidarity. The tendency to favor the ingroup over the outgroup and to think of the world in ‘us versus them’ terms is known to affect several political and social attitudes (31). Using Link and Phelan (32) conceptualization of stigma, the ‘us versus them’ thinking (i.e., the cognitive separating component) is also associated with negative attributions to the outgroup and social distancing. People with a mental illness are perceived as distinct and separate from those without mental illness. This ‘us versus them’ mentality fosters the belief that people with mental illnesses are fundamentally different, thereby increasing social distance (32, 33).

Regarding the desire for social distance from people with depression or schizophrenia, we found significant milieu differences, whereby the desire for social distance from people with schizophrenia was generally more pronounced than the desire for social distance from people with depression. This finding is in line with previous research (8, 11, 34) arguing that firstly, the acceptance of biological explanations for serious mental illnesses increases stigmatizing attitudes and social distance, secondly, buffering factors such as familiarity with the disorder due to own clinical history or of that of a close other are more strongly related to depression than in schizophrenia, and thirdly, depression is more likely viewed in a dimensional versus a categorical conceptualization (8).

On milieu level, our findings suggest that milieu groups with a more liberal attitude on the socio-cultural dimension expressed a lower desire for social distancing towards depression and there was also a certain tendency to express a lower desire for social distancing towards schizophrenia. Regarding depression, distinctiveness (i.e., based on the number of statistically significant mean differences between the more liberal and the more authoritarian milieu groups) was high. Significant mean differences were detected between liberal – and all authoritarian milieu groups (i.e., *the cosmopolitan intellectuals*) or at least four out of six authoritarian milieu groups (i.e., *the committed citizenship, the social market optimists*). Those who speak out in favor of social liberalization, but also endorse economic conservatism (*the cosmopolitan intellectuals, the committed citizenship)* desired less social distance from the people depicted in the vignettes as well as those who speak out of in favor of both, social liberalization, and market liberalization *(the social market optimists)*. Social conservatism or authoritarianism and intolerance of deviance or outgroups are often viewed as intertwined, as well as tolerance and liberalism (35). Tolerance is usually regarded as an integral part of the liberal identity and probably both are consequences of personal traits like openness.

Several studies have shown that openness may influence prejudices and tolerance (36) and beyond that, several other studies have already shown that openness to experiences is positively correlated with liberalism (37). It is therefore quite possible that milieu groups with a more liberal attitude on the socio-cultural dimension (i.e., *the cosmopolitan intellectuals, the committed citizenship*, and *the social market optimists)* are more open to mental health issues and therefore express a lower desire for social distance towards people with mental illnesses.

Research further reveals that liberals want to feel more empathy and experience more empathy than conservatives do (38). They might more often talk and listen to stories of lived experiences, which in turn can break down the stigma surrounding mental illnesses. Further, consensus within the two poles with more liberal – or authoritarian values was high. For example, we identified only one significant pairwise comparison within all authoritarian milieu groups with *the disappointed* having the highest mean scores on the SDS and *the market sceptics* having the lowest mean scores. Those who voice doubts and disappointment about a lack of fairness, societal diversity, and European solidarity (i.e., *the disappointed*) are more negative towards people with depression depicted in the unlabeled vignettes. The doubts and uncertainties regarding the (unpredictable) dynamics of the world seem to be a basic characteristic of this milieu group and the desire for social distance might be a further consequence of increased uncertainty regarding the deviation from ideal or normal mental health. The unpredictability – not only regarding the individual with depression in the case vignettes but also regarding political, social, or economic changes across the world – potentially raises several doubts and uncertainties which in turn might lead to people withdrawing or wanting more distance.

Regarding schizophrenia, distinctiveness between the more liberal – and the more authoritarian milieu groups was less pronounced. Notably, a smaller number of significant mean differences was observed. Particularly for *the social market optimists*, we observed only two significant mean differences and that was between milieu groups with the highest (i.e., *the conservatives*) and the second highest (i.e., *the participation-oriented*) preferences for social distance. Compared to the depression vignette, consensus within the more authoritarian milieu groups was comparable. We identified two significant pairwise comparisons within all authoritarian milieu groups, located all within the fourth quadrant of the two-axis chart (i.e., representing authoritarian values and economically liberal positions). Those, who emphasize individual performance, performance-related compensation, and gratification by opposing social assistance transfers as well as preferring a profit-oriented economy (i.e., *the conservatives*) more intensively wanted to distance themselves from people with schizophrenia than milieu groups who emphasize even more the benefits of market liberalization and competition (i.e., *the performance-oriented)* or adopt even more authoritarian values (i.e., *the individualists*). In contrast to the third quadrant of the two-axis chart (i.e., representing authoritarian values and economically conservative positions), the fourth quadrant appears to be more inhomogeneous and predominantly more tolerant towards people with schizophrenia. The preferences of *the performance-oriented* were similar to the preferences of all three milieu groups with a more liberal attitude. An almost similar pattern was observed for *the individualists*. Only *the conservative’s milieu group*, with a larger proportion of older men with higher incomes, does not seem to fit the pattern here. These ‘successful’ high performers diverge from predominantly female and more liberal milieu groups as well as lower-income milieu groups within the fourth quadrant (i.e., *the performance-oriented* and *the individualists*). For *the conservatives*, having a serious mental illness such as schizophrenia might be more associated with failure and poor performance and therefore incompatible with their own self-conception.

This would also fit into the picture that *the conservatives* themselves are less likely to seek psychotherapeutic help due to mental health problems. Their understanding of mental illnesses might diverge from prevailing ideas of masculinity and performance expectations (26) which in turn could also be evident in their dealing with people with schizophrenia (39).

Overall, our findings suggest that milieu-specific differences are more visible regarding depression than schizophrenia. We discussed a higher liberal-authoritarian distinctiveness regarding the desire for social distance towards people with depression than towards people with schizophrenia. Explaining this pattern is actually somewhat difficult; however, we argue that the manner in which mental health awareness campaigns are designed and delivered could be used as an explanation. Awareness campaigns primarily refer to depression and less to schizophrenia. Consequently, primarily attitudes towards people with depression are changing (40). At the same time, research (41) finds that there are demographic groups with higher campaign awareness (e.g., women, younger people). We argue that people with more liberal attitudes may also be more receptive to destigmatizing messages about depression but not about schizophrenia. Schizophrenia is usually not visible in awareness campaigns. Across all milieu groups, there is a general level of unfamiliarity. Milieu-specific differences are therefore less visible.

The year 2024 marks more than 30 years since the WHO systematically addressed the stigma related to mental illness and its consequences through awareness raising and advocacy efforts. In contrast to the 1990s, awareness of (certain) mental illnesses is on the rise, and much has been done to achieve the goal of ending stigmatization.

Stigmatization has been seen through the lenses of different perspectives and countries (for an overview see 42). However, there is still much to be done. According to this, a Lancet commission on ending stigma and discrimination in mental health has summarized interventions for stigma reduction. In this umbrella review of 216 systematic reviews, interventions based on the principle of social contact that have been appropriately adapted to different contexts and cultures are highlighted as particularly effective to reduce stigmatization worldwide (for an overview see 43). Milieu research typically provides insights into the context of individuals’ lives and therefore recognizes the social embeddedness of stigmatization.

### 4.1 Conclusion

As a result, we illustrate that milieu groups with similar liberal or authoritarian values do not automatically represent similar preferences related to mental illnesses. For a better understanding, a distinction between a cultural liberal-authoritarian dimension and a socio-economic dimension (market liberalization vs. a more regulated economy) therefore provide an additional explanatory power. Milieu-specific knowledge could be useful in deciding which intervention components are most appropriate for which milieu groups (e.g., psychoeducation, social contact, social networking). There seem to be milieu groups in which negative attitudes towards people with mental illnesses seem to be more pronounced than in other milieu groups (e.g., *the participation-oriented)* as well as milieu groups with a stronger desire for social distance from people with serious mental illness (e.g., *the conservatives*). Moreover, there is need to understand how to apply a milieu-specific lens to specific interventions. This includes not only the consideration of specific components or techniques to reduce stigma, but also the way in which groups can be addressed in a milieu-specific way. Building on previous works (e.g., the recommendation of the Lancet Commission) but setting milieu-specific foci and making blind spots visible (i.e. schizophrenia) might be useful to combat stigmatization towards people with serious mental illnesses.

### 4.2 Strengths and limitations

In the present study, we examined stigmatization towards people with mental illnesses at latent group level by considering socioeconomic and socio-cultural value differences. We therefore combined traditional knowledge on public’s attitudes and preferences for social distance from people with depression or schizophrenia with additional contextual knowledge. This broader approach provides a more contextualized understanding of stigmatization in mental illnesses, and it might be this ‘contextualized lens’ on stigmatization that will aid in developing more effective anti-stigma interventions. Our study also has some limitations. Since no standard typology of milieus in Germany exists, and several limitations are discussed (e.g., the conceptualizing of values, the consideration of socio-economic characteristics or the replicability) (44), a milieu-typology already used for policy, research, and debates on right-wing populism (25) was chosen for the present study. While we have utilized values and political preferences that are probably not limited to the German context, milieu typologies are generally specific to the context in which they were developed. Consequently, the applicability of these findings to other countries remains uncertain. Related to preferences of social distance from people with mental illnesses, we focused on the ‘would do’ in hypothetical situations. However, we cannot draw conclusions on actual behavior in real-life from this.

## Data Availability

The raw data supporting the conclusion of this article is available from the authors upon request.
