# Supplementary material for: Stigmatization of people with mental illness – a matter of milieu-specific worldviews? Results from a population-based survey in Germany
Source: Front Psychiatry. 2025 Jan 28;15:1501194. doi: 10.3389/fpsyt.2024.1501194 (PMC11810944; doi:10.3389/fpsyt.2024.1501194)
Supplement: Supplementary file 1 [file Table1.docx]

Supplementary Material

**Stigmatization of People with Mental Illness – A Matter of Milieu-Specific Worldviews? Results from a Population-Based Survey in Germany.**

**J Spahlholz, E Baumann, S Speerforck, C Sander, M C Angermeyer & G Schomerus**

***Correspondence:** Jenny Spahlholz: jenny.spahlholz@medizin.uni-leipzig.de

***Supplementary Table 1.*** Sample Characteristics (N=3,042)

|  | Sample  (n=3,042)  % |  | German general adult population^a^ |
| --- | --- | --- | --- |
|  |  |  |  |
| Age mean (SD) | 49.17 (17.35) |  | - |
| Age-groups |  |  |  |
| 18-25 years | 10.4 |  | 10.4 |
| 26-45 years | 32.3 |  | 29.9 |
| 46-60 years | 28.5 |  | 27.3 |
| >60 years | 28.9 |  | 32.4 |
|  |  |  |  |
| Gender |  |  |  |
| Female | 52.4 |  | 50.7 |
| Male | 47.2 |  | 49.3 |
| Divers | 0.4 |  |  |
|  |  |  |  |
|  |  |  |  |
| Education |  |  |  |
| Unknown/pupil | 1.0 |  | 0.4 |
| No schooling completed | 1.6 |  | 4.1 |
| 8-9 years | 28.3 |  | 29.8 |
| 10 years | 41.1 |  | 30.7 |
| 12-13 years | 28.0 |  | 34.8 |
|  |  |  |  |
| Income^b^ |  |  |  |
| <2,000 € | 39,9 |  | n.a. |
| 2,000-2,500 € | 16,1 |  | n.a. |
| 2,500-3,500 € | 27,4 |  | n.a. |
| >3,500 € | 16,6 |  | n.a. |
|  |  |  |  |
| Milieu groups |  |  |  |
| Committed citizenship | 14.0 |  | - |
| Cosmopolitan intellectuals | 11.7 |  | - |
| Conservatives | 11.7 |  | - |
| Social market optimists | 11.0 |  | - |
| Performance-oriented | 6.8 |  | - |
| Individuals | 17.1 |  | - |
| Disappointed | 13.0 |  | - |
| Market-sceptics | 10.9 |  | - |
| Participation-oriented | 3.9 |  | - |
| *Note.* ^a^Reference values from German Federal Statistics Office (2019), ^b^Comparable data on household income was not available. Total household income was assessed without taking the number of household members into account; n.a.=not available | | | |
